# Diabetic Hand: A Neglected Complication of Diabetes Mellitus

**DOI:** 10.7759/cureus.2772

**Published:** 2018-06-09

**Authors:** Alpesh Goyal, Vivek Tiwari, Yashdeep Gupta

**Affiliations:** 1 Endocrinology and Metabolism, All India Institute of Medical Sciences, New Delhi, IND; 2 Department of Orthopaedics, All India Institute of Medical Sciences, New Delhi, IND; 3 Department of Endocrinology and Metabolism, All India Institute of Medical Sciences, New Delhi, IND

**Keywords:** limited joint mobility, diabetic hand, cheiroarthropathy, dupuytren's disease

## Abstract

Diabetes mellitus is associated with a variety of musculoskeletal (MSK) manifestations affecting the hand, which can significantly affect a patient’s quality of life. While a great deal of attention is paid to the chronic microvascular complications of diabetes, the MSK complications are often ignored in clinical practice. It is important to diagnose them as their presence has been found to correlate with chronic microvascular complications of diabetes especially retinopathy. We describe a case of a young male with long-standing type 1 diabetes mellitus and chronic microvascular complications, who presented to us with several manifestations of diabetic hand syndrome.

## Introduction

Diabetes mellitus is associated with a constellation of debilitating musculoskeletal (MSK) disorders affecting hand, commonly referred to as diabetic hand syndrome. These include limited joint mobility (LJM) (also known as diabetic cheiroarthropathy), Dupuytren’s contracture, stenosing tenosynovitis (trigger finger), carpal tunnel syndrome (CTS), Charcot neuroarthropathy, reflex sympathetic dystrophy and a variety of hand infections which these individuals are predisposed to [1-3]. Timely recognition of these conditions not only helps in instituting proper treatment to decrease morbidity and disability, but also aids in early identification of vascular complications of diabetes which are found to be related to the hand symptoms [4].

## Case presentation

**Figure 1:**
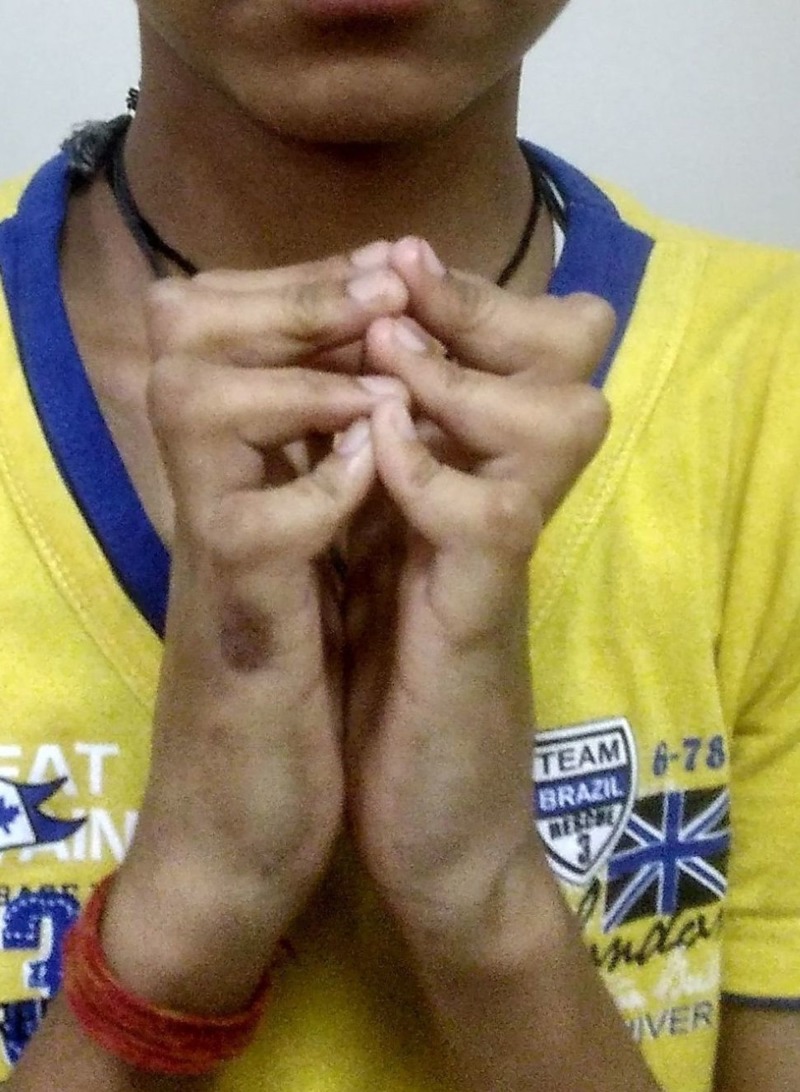
Positive prayer sign. Positive prayer sign with fixed flexion deformity at proximal interphalangeal joints suggestive of Limited Joint Mobility.

**Figure 2:**
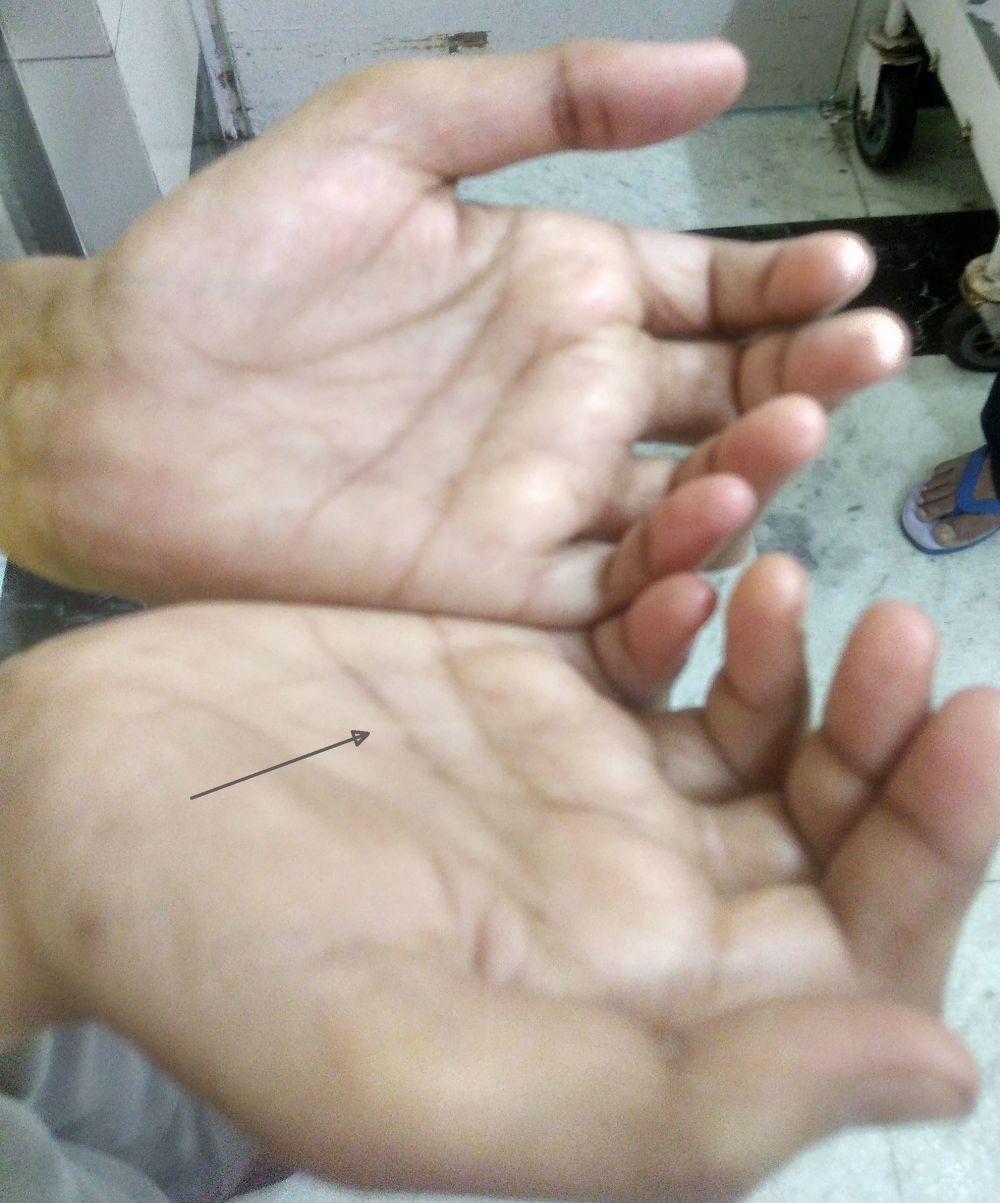
Dupuytren's contracture. Nodular thickening and induration of palms suggestive of Dupuytren’s contracture (black arrow).

**Figure 3:**
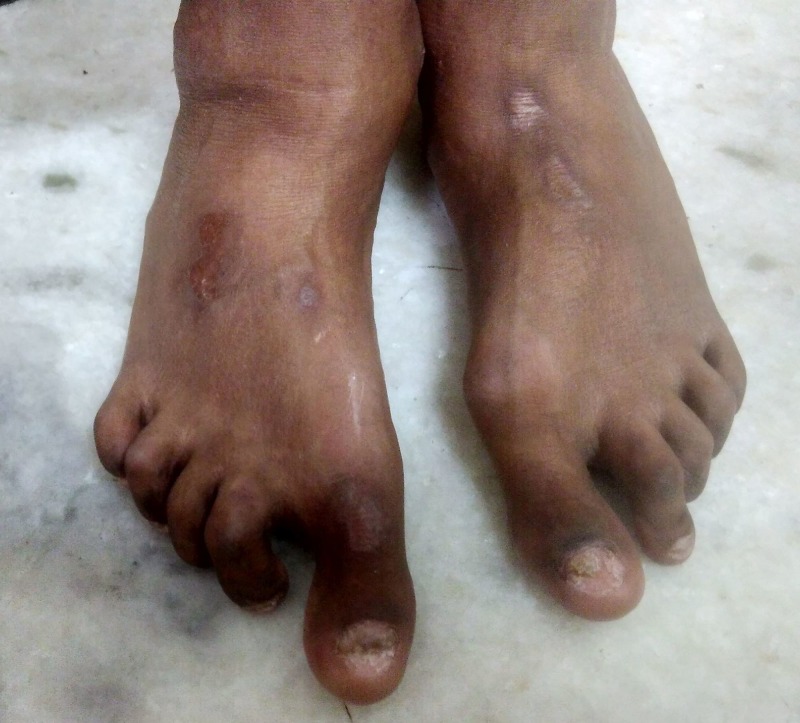
Foot deformities. Bilateral deformed foot with hammer toes and hallux valgus.

A 20-year-old male patient, with type 1 diabetes mellitus of 15 years duration, on twice daily premixed insulin and poor glycemic control (glycated hemoglobin of 10.8%), presented to us with evidence of advanced microvascular disease. He had bilateral proliferative diabetic retinopathy (PDR), distal symmetrical sensorimotor polyneuropathy (DSSN), autonomic neuropathy and nephrotic range proteinuria with new onset hypertension without azotemia. He had high-risk bilateral foot with the presence of hammer toes and hallux valgus but without any active foot ulceration. Hand examination revealed fixed flexion deformity at proximal interphalangeal joints with associated tightening of the skin and cord-like induration on palms at the level of metacarpophalangeal joints (Figures 1-3).

Tinel’s sign and Phalen’s test (for carpal tunnel syndrome) were negative and there was no evidence of trigger finger. Furthermore, there was no finding suggestive of adhesive capsulitis of the shoulders. He denied any history of joint pain, swelling, early morning stiffness, Raynaud’s phenomenon or local trauma. The patient, however, did not have any functional limitation associated with the hand deformities as he could inject insulin, use computer keyboard and carry out all his routine work without any difficulty. Radiographs of hands and feet were negative for any neuroarthropathy or inflammatory arthropathy; immunological markers were also negative. In the background of chronic longstanding diabetes with poor glycemic control and advanced microvascular complication, diagnosis of diabetic hand syndrome was entertained.

The patient was shifted to multiple subcutaneous insulin injection (basal and bolus regimen) for better glycemic control and blood pressure control achieved using renin-angiotensin-aldosterone (RAAS) blockade combined with calcium channel blocker. For PDR, the patient underwent the first session of pan-retinal photocoagulation (PRP). He was provided with customized footwear and educated regarding foot care for the high-risk feet. For hand deformity, hand surgeon was consulted, who advised for conservative management in the form of stretching exercises and braces in view of minimal functional disability and guarded results of the surgery.

## Discussion

Diabetic hand syndrome is a common, yet relatively less discussed entity. Pathologies described under the umbrella of diabetic hand syndrome occur in general population as well; however, they are more common in patients with diabetes. These may differ in their mode of presentation, natural course and treatment response in diabetics compared to normal population. This condition has also been referred to as diabetic pseudoscleroderma in literature and when combined with adhesive capsulitis of the glenohumeral joint, the term shoulder-hand syndrome is often used [1-3]. Our patient had manifestations of diabetic hand syndrome in the form of LJM and Dupuytren’s contracture. An inflammatory arthropathy or connective tissue disorder as the cause for joint deformities and skin tightening were excluded with relevant investigations. His glycemic control was optimized and conservative management preferred in view of minimal functional disability.

LJM is a frequently overlooked hand complication of diabetes mellitus; it is more common in patients with longstanding and uncontrolled disease with prevalence varying from 8 to 50%. Typically, it presents with the limitation of extension of the metacarpophalangeal, proximal interphalangeal, and distal interphalangeal joints, starting on ulnar side and spreading radially [5]. It can easily be diagnosed by eliciting simple bedside signs such as “prayer sign” or "table top sign". Prayer sign is elicited by asking the patient to hold the hands in opposition to one another vertically, keeping the elbows flexed and wrists extended. If the patient is unable to completely approximate the palmar surface of the corresponding digits, the sign is considered positive. Table top sign is elicited by asking the patient to place the palms flat on a hard surface such as a table and inspecting if the entire palmar surface of the digits touches the table. A positive sign is indicated by the inability to make the digits and palm lie flat on the table. Dupuytren’s contracture is characterized by contractures of digits, most commonly involving the middle and ring fingers in diabetics, along with nodular thickening of palmar skin with associated tethering. Pathophysiology includes the formation of advanced glycation end products, cross-linking of collagen, with the formation of stiff collagen which is resistant to degradation, and microangiopathy affecting the skin and subcutaneous vessels [6]. Other hand conditions which are more common in diabetic population than general population include carpal tunnel syndrome and stenosing tenosynovitis (trigger finger).

In previous studies by Rosenbloom et al. [7] and Lawson et al. [8], examining the relationship between hand manifestations and chronic microvascular complications of diabetes, LJM was found to be related to diabetic retinopathy. In another study by Comi et al. [9], carpal tunnel syndrome was found to be correlated independently to retinopathy, peripheral neuropathy, stenosing tenosynovitis, and Dupuytren’s contracture in diabetic population.

Treatment for these conditions is associated with substantial improvement in the quality of life. Achievement of good glycemic control can not only help in prevention but can also reverse the musculoskeletal complications early in the natural history. Specific orthopedic interventions may be indicated in some cases. However, clinicians should be aware that specific treatment response such as results of carpal tunnel release surgery for CTS and steroid injection for trigger finger may be worse in diabetics compared to the general population.

## Conclusions

We have highlighted the importance of simple clinical examination in diagnosing various MSK manifestations of diabetes mellitus. The hand manifestation of diabetes mellitus may serve as a mirror for microvascular complications like retinopathy. Achievement of optimal glycemic control and specific orthopedic interventions in selected cases are the mainstay of management in these cases.
